# Structure-based metabolite function prediction using graph neural networks

**DOI:** 10.1093/bioadv/vbaf174

**Published:** 2025-07-21

**Authors:** Tancredi Cogne, Mariam Ait Oumelloul, Ali Saadat, Janna Hastings, Jacques Fellay

**Affiliations:** School of Life Sciences, Ecole Polytechnique Fédérale de Lausanne, Lausanne, 1015, Switzerland; School of Life Sciences, Ecole Polytechnique Fédérale de Lausanne, Lausanne, 1015, Switzerland; Swiss Institute of Bioinformatics, Lausanne, 1015, Switzerland; School of Life Sciences, Ecole Polytechnique Fédérale de Lausanne, Lausanne, 1015, Switzerland; Swiss Institute of Bioinformatics, Lausanne, 1015, Switzerland; Swiss Institute of Bioinformatics, Lausanne, 1015, Switzerland; Institute for Implementation Science in Health Care, Faculty of Medicine, University of Zurich, Zurich, 8006, Switzerland; School of Medicine, University of St. Gallen, St. Gallen, 9000, Switzerland; School of Life Sciences, Ecole Polytechnique Fédérale de Lausanne, Lausanne, 1015, Switzerland; Swiss Institute of Bioinformatics, Lausanne, 1015, Switzerland; Biomedical Data Science Center, Lausanne University Hospital and University of Lausanne, Lausanne, 1011, Switzerland

## Abstract

**Motivation:**

Being able to broadly predict the function of novel metabolites based on their structures has applications in systems biology, environmental monitoring, and drug discovery. To date, machine learning models aiming to predict functional characteristics of metabolites have largely been limited in scope to predicting single functions, or only a small number of functions simultaneously.

**Results:**

Using the Human Metabolome Database as a source for a wider range of functional annotations, we assess the feasibility of predicting metabolite functions more broadly, as defined by four elements, namely location, role, the process it is involved in, and its physiological effect. We evaluated three graph neural network architectures to predict available functional ontology terms. We compared the graph models with two multilayer perceptron architectures using circular fingerprints and Chemical BiDirectional Encoder Representations from Transformers (ChemBERTa) embeddings. Among the models tested, the graph attention network, incorporating embeddings from the pretrained ChemBERTa model to predict the process metabolites are involved in, achieved the highest performance with a macro F1-score of 0.903 and an area under the precision-recall curve of 0.926.

**Availability and implementation:**

The model identified function-associated structural patterns within metabolite families, demonstrating the potential for interpretable prediction of metabolite functions from structural information.

## Introduction

Metabolites are the small molecules produced during metabolism that play essential roles in biochemical pathways in all living organisms. Understanding their functions is important to advance the fundamental understanding of molecular and cellular pathways and for a range of different application areas including environmental monitoring and drug discovery. Functions are commonly linked to structures in biochemistry, including for metabolites (Nobeli *et al.* 2003, Hatzimanikatis *et al.* 2004), enabling the potential prediction of functional characteristics based on structures. This idea has been extensively explored for proteins, for which the prediction of function based on structure is a longstanding grand challenge in computational biology (Cruz *et al.* 2017, Kulmanov *et al.* 2018, Zhao *et al.* 2021, Lai and Xu 2022). Protein function prediction has seen great improvements in recent years with the rise of machine learning models such as DeepFRI (Gligorijević *et al.* 2021), which predicts functional gene ontology (GO) terms based on the structure of proteins.

To our knowledge, unlike for proteins, for metabolites no machine learning model exists to predict a broad range of functions at the same time based on their structure. A possible explanation could be the lack of a gold standard ontology for metabolites that matches the scale of the GO for proteins. Various ontologies exist, such as the “role” branch of the ChEBI (Degtyarenko *et al.* 2008) ontology or ChemFOnt (Wishart *et al.* 2023), but these are not as complete as GO or as comprehensive in terms of annotations. Nevertheless, chemical compounds have more stable and defined structures than proteins, which should represent a valuable source of information. An increase in the number and scale of publicly accessible metabolite databases, such as the Human Metabolome Database (HMDB) (Wishart *et al.* 2022), provides an opportunity to address this gap.

We aimed to develop a model to address the challenge of predicting multiple metabolite functions at the same time based on their structure. We hypothesize that the molecular structures of metabolites contain sufficient information to enable prediction of their functional metabolic characteristics as defined by their location, their role, the processes they are involved in, and their physiological effects (as per HMDB annotations). Based on the logical representation of molecules as graphs, we compare three different graph neural network (GNN) architectures for this task as well as two multilayer perceptron (MLP) models using circular fingerprints (baseline model) and Chemical BiDirectional Encoder Representations from Transformers (ChemBERTa) embeddings. Furthermore, we show the ability of an attention-based model to detect the importance of certain molecular substructures for the function of molecules.

### Related work

Advancements in protein function prediction have been driven by machine learning models that effectively leverage sequence and structural information. For example, DeepGO uses deep learning and protein sequence embeddings to predict protein function annotations, with its updated version, DeepGO-SE, incorporating a pretrained large language model (LLM) to predict GO functions (Kulmanov *et al.* 2024). Similarly, DeepFRI combines graph convolutional networks (GCNs) and protein contact maps to identify functional sites in protein structures. GCNFold (Enbin *et al.* 2023) applies GCNs to protein structure graphs for functional site prediction, while TAWFN integrates convolutional neural networks and GCNs for enhanced protein function annotation (Meng and Wang 2024).

While these advances highlight the power of machine learning in protein function prediction, no model has yet been developed to perform multilabel functional predictions specifically for metabolites. In the broader domain of chemical predictive modelling, Quantitative Structure–Activity Relationship (QSAR) (Soares *et al.* 2022) models link molecular structure to specific biological activities, e.g. models may predict binding affinity, inhibitory concentration, or toxicity using linear and nonlinear methods. Other models have been developed to address related prediction tasks, such as Disease and Literature driven Metabolism Prediction Model (DLMPM) (Wang *et al.* 2022a), which employs a latent factor model to identify disease-metabolite associations, and Disease Related Metabolites (Deep-DRM), which applies graph deep learning techniques for the same purpose. Graph Convolutional Network with graph Attention Network (GCNAT) (Sun *et al.* 2022) combines GCNs with graph attention networks, again for predicting disease-metabolite associations. Expanding to molecular property prediction, Chen et al. (2024) used GNNs to predict properties such as boiling points and mass spectra. Huckvale and Moseley (2024) proposed a model to determine metabolite-pathway involvement using features of both metabolites and pathways. Additionally, Porokhin et al. (2023) demonstrated GNN applications for site-of-metabolism prediction, while Glauer et al. (2024) introduced a transformer-based model to extend ChEBI’s ontology (the structural branch rather than the role branch) by classifying unseen chemical structures.

Finally, ChemBERTa (Chithrananda *et al.* 2020) can learn molecular representations using Simplified Molecular Input Line Entry System (SMILES) and was used in our model to improve the results. Molecular Contrastive Learning of Representations (MolCLR) (Wang *et al.* 2022b) also aims to learn molecular representations in a self-supervised manner.

### Contributions of our study

We propose a novel machine learning approach for predicting multiple metabolite functions based solely on chemical structure, addressing a significant unmet need in current metabolomics research, where increasingly large numbers of metabolite structures may be characterised in samples yet not be associated with any functional annotations that might support their functional interpretation.

We extract and filter a dataset of 3278 metabolite structures and associated functions from the HMDB. We evaluated and compared three GNN architectures: GCN, graph isomorphism network (GIN), and Graph Attention Network (GAT), while also assessing the effectiveness of ChemBERTa embeddings to augment these models. We compared performance with two MLP models using circular fingerprints (baseline model) and ChemBERTa embeddings. We highlight the attention-based model’s ability to detect important molecular substructures by leveraging explainable AI techniques on the attention weights.

## Methods

### Database

The HMDB, released on 2 November 2021, includes 217,920 metabolites, each characterized by various attributes such as molecular weight and chemical structure. The HMDB’s functional hierarchical structure comprises 2009 distinct ontology terms, all of which are categorized under one of four primary nodes:

- “Disposition” is defined by the HMDB as the “origin of a chemical, its location within an organism or its route of exposure.” We will refer to this category as “location.”
- “Role” is defined by the HMDB as the “purpose or function of a chemical, either naturally or as intended by humans.”
- “Process” is defined by the HMDB as the “biological or chemical events, or a series thereof, leading to a known function or a known end product.”
- “Physiological effect” is defined by the HMDB as the “measured or observed physiological effect on an organism resulting from its exposure to a chemical.”

### Metabolite filtering

**Figure 1.**
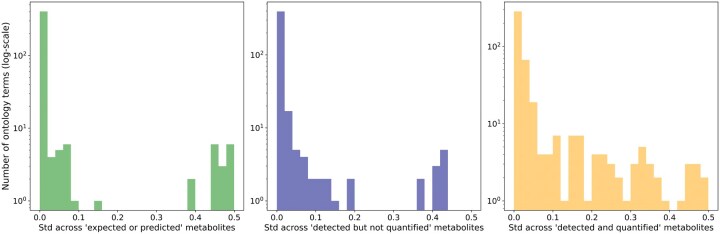
Histogram of standard deviation across the different categories of metabolites as described in the HMDB. Many ontology terms have a near zero standard deviation and were filtered out to retain only a small subset of ontology terms as outputs of the model. Distribution for all the “expected” or “predicted” metabolites (green), “detected but not quantified” metabolites (blue), and “detected and quantified” metabolites (yellow).

Each metabolite in HMDB falls into one of four categories: “expected,” “predicted,” “detected but not quantified,” or “detected and quantified.” For each ontology term, we counted the number of metabolites associated with it, allowing us to calculate the standard deviation across different metabolite categories (Fig. 1) using:


$$\sigma_{j}=\sqrt{\frac{\sum_{i}{\left(x_{i,j}-{\bar{x}}_{j}\right)}^{2}}{N-1}}$$

Here, $x_{i,j}$ represents the binary value for metabolite *i* and ontology term *j*. ${\bar{x}}_{j}$ is the mean value of the binary values for ontology term *j* and *N* is the number of metabolites. This analysis was based on the assumption that if all metabolites are associated with a given term, or none are, the standard deviation would be zero, indicating a lack of informative value for the model. An important difference can be seen in the different distributions. Most ontology terms have a near zero standard deviation when looking only at “expected,” “predicted,” or “detected but not quantified” metabolites. This can be explained by the fact that most of them do not contain any ontology-related information and thus are set to “false,” yielding a very small standard deviation. Thus, we decided to focus on the “detected and quantified” (3278 metabolites) category (Fig. 1) because it was the only one with enough ontology-related information to test our hypothesis.

### Label filtering

**Figure 2.**
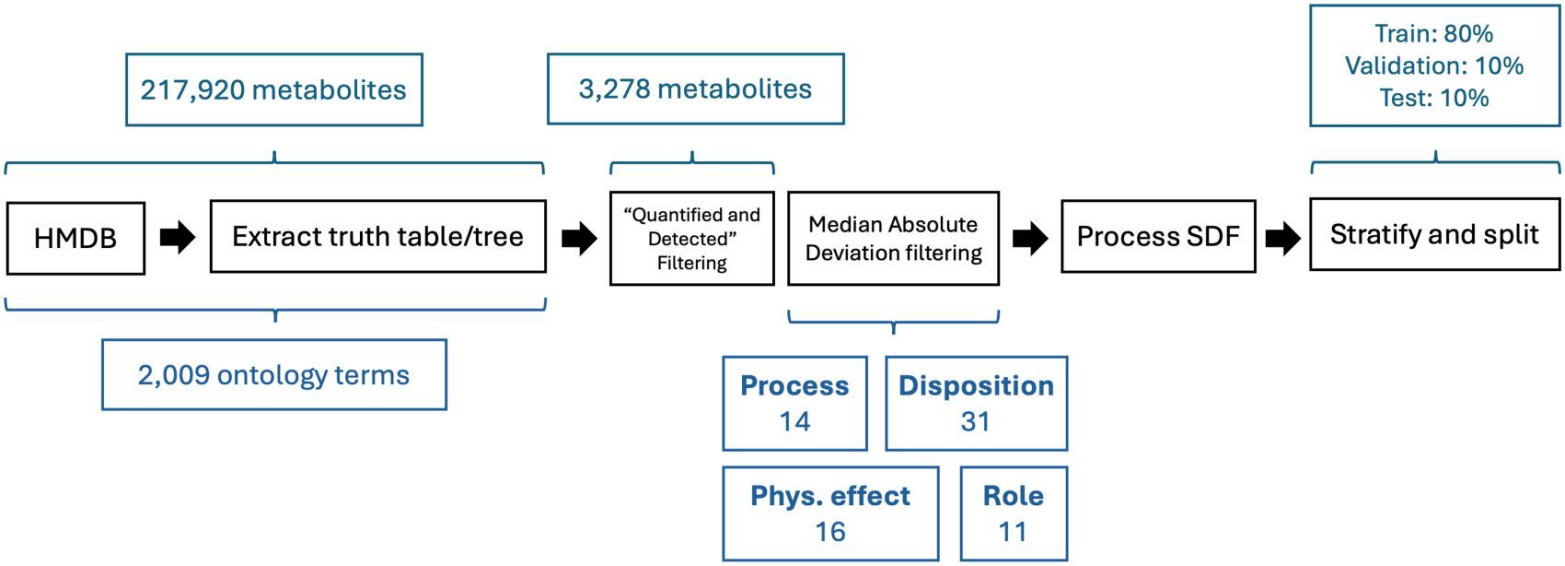
Data processing pipeline. Files from the HMDB were parsed to extract a truth table and a tree ontology. Metabolites were filtered by keeping only the ones with the label “detected and quantified.” Ontology terms were first filtered to keep only the terminal (without a child) nodes. A subset was selected using median absolute deviation filtering. Input/output information was extracted for this given set of metabolites and ontology terms. The data was stratified and split to tackle the unbalanced nature of the data set regarding possible outputs.

We used median absolute deviation (MAD) to filter which ontology terms were used as an output of the model. MAD is a robust statistical measure of variability and is sensitive to outliers. The threshold was selected with a modified Z-score $M_{i}$ based on the similarity with a gamma distribution (Itan *et al.* 2015):


$$M_{i}=\frac{0.6745(s_{i}-s)}{\text{MAD}}$$

where $s_{i}$ is the standard deviation of the ontology term *i*, *s* is the median standard deviation, and MAD is the median absolute deviation. Terms with an absolute $M_{i}>3.5$ were selected as outputs of the model. After filtering, 14 child nodes remained in “Process,” 31 in “Disposition,” 16 in “Physiological effect,” and 11 in “Role” (Fig. 2). MAD filtering allowed us to select which labels to focus on. Nevertheless, some selected labels are exhibited less frequently than others by metabolites, and thus might be more prone to type II errors (false negatives) (Fig. S1). This imbalance may have some impact on the performance, and we have therefore used a panel of metrics to account for this possibility.

### Data processing

**Figure 3.**
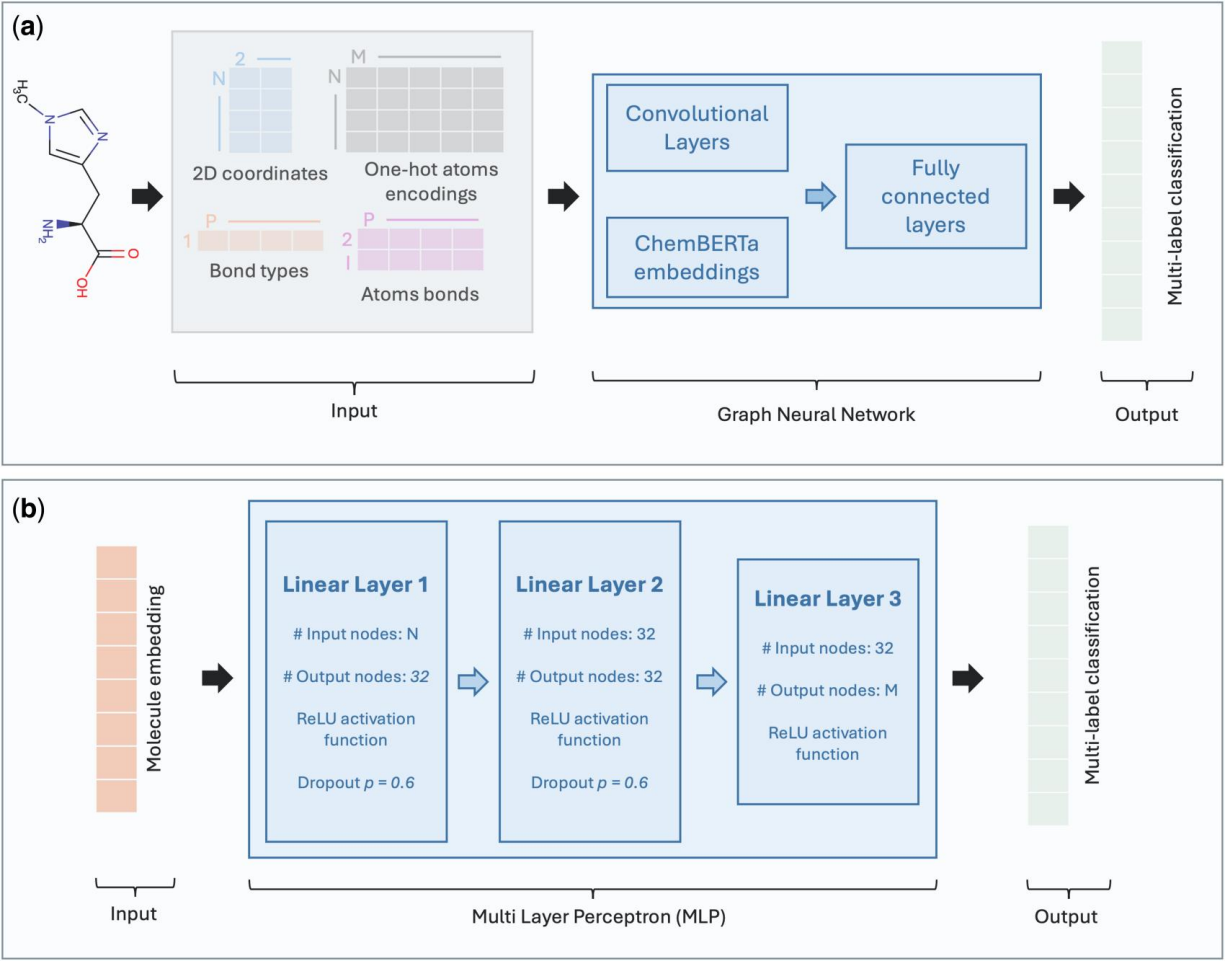
Visual representation of the models from input to output. Multilabel classification is used and a binary value is outputted for each selected ontology term. (a) Graph models. *N* is the number of atoms in a given metabolite, *M* the number of unique atoms across all metabolites, and *P* is the number of bonds of a given metabolite. The input is composed of four arrays to describe all the parts of each molecule: 2D coordinates (N×2), atomic numbers (N×M), and bonds (1×P and 2×P). The compared architectures all include convolutional layers followed by fully connected layers. (b) MLP models. *N* is the length of the inputs (1024 for circular fingerprints and 600 for ChemBERTA embeddings), *M* is the number of output nodes (which is different for “Disposition,” “Role,” “Process,” and “Physiological effect”). The molecule embedding corresponds to circular fingerprint for the baseline model and to ChemBERTA embedding for the MLP model. The two MLP architectures both include three linear layers.

Graph representation is commonly used for molecules (Wu *et al.* 2018, Colliandre, 2020, Daigavane *et al.* 2021, Raghunathan and Priyakumar 2022, Song *et al.* 2023) as a direct mapping can be made by using a node for each atom and an edge between two atoms for each bond. The input representation used in our model stores multiple pieces of information for each metabolite: 2D coordinates as given by the HMDB and atomic number of each atom, the two atoms at the extremities of each bond, and the bond type (single, double, …). One-hot encoding is commonly used for categories in machine learning to avoid arbitrary ordinal relationships and was used in this model to represent each possible atom of a metabolite. This resulted in the array *A* of shape N×M where *N* is the number of atoms in a given metabolite and *M* the number of unique atoms (Fig. 3a). Each column corresponds to a specific chemical element. $A_{i,j}$ is equal to 1 if the ith atom of the metabolite is the chemical element corresponding to the one in column *j*. Each row can thus only have one single non-zero value. The 2D coordinates of each molecule were normalized and standardized, as given that one-hot encodings were also used as input of the model, having both extreme coordinates and one-hot encodings as inputs would result in the model likely focusing only on the coordinates. The model predicts a binary value separately for each selected ontology term (multilabel classification). Even though some terms are related, the model treats all outputs as independent for simplification.

Due to the small size and the unbalanced nature of the dataset, the split between training and test sets had to be carefully done. Indeed, many metabolites share similar outputs. To avoid their uneven grouping in the training (or test) sets, the metabolites were clustered based on output. Each cluster was split with a ratio of 0.9/0.1. This makes sure that every label will be present in both training and test sets.

### Architectures

We used three graph architectures using convolutions and compared their performance: GCN, GIN, and GAT (Elton *et al.* 2019, Sanchez-Lengeling *et al.* 2021). GCNs extend the concept of convolution from grid-like data (such as images) to graph data, allowing the aggregation of feature information from neighboring nodes. This approach effectively captures local graph structure and node features. GINs are designed to be powerful for graph isomorphism, making them capable of distinguishing a wide variety of graph structures. They achieve this by using a MLP to aggregate node features, enhancing their discriminative power. GATs introduce an attention mechanism to GNNs, enabling nodes to assign different importance weights to their neighbors. This allows for more flexible and expressive feature aggregation, potentially improving performance on tasks where certain neighbors have more influence than others. Each architecture was made of two convolutional layers followed by a fully connected layer (Fig. 3a and Fig. S2).

To assess the importance of the graph representation, we included two MLP models including a baseline. They both are composed of three linear layers (Fig. 3b). The baseline model’s inputs are circular fingerprints generated by RDKit from the molecule’s SMILES. The second MLP model uses ChemBERTa embeddings as inputs.

The Adam optimizer with the binary cross entropy loss was used for all the models. Sum aggregation was used to aggregate the features in order to do graph classification.

### Embedding calculation

To improve model performance, pretrained models are commonly used to include additional information. We tested this strategy with the pretrained model ChemBERTa (Ahmad *et al.* 2022), which is inspired by the BiDirectional Encoder Representations from Transformers (BERT) (Devlin *et al.* 2019) LLM applied to molecules. This chemical model was trained on 77M unique SMILES annotations from PubChem (Kim *et al.* 2019). For each part of the SMILES, an embedding, i.e. a vector capturing important semantic and structural features of the molecule, is obtained. By averaging these embeddings, we obtained a vector representation of each molecule which is added before the fully connected layer of the model (Fig. 3a).

### Metrics

Due to the limited size of the dataset used (3278 metabolites) and the small proportion of metabolites with specific ontology terms, the model is susceptible to generate a high number of false negatives. Accuracy is thus not a suitable metric to evaluate the model. We used instead the macro F1-score, which combines precision and recall. The true positive rate, also known as recall, was also used since we were mainly interested in knowing which ontology terms are associated with a given metabolite. To further assess performance, we included weighted F1-score and the Area Under the Precision-Recall Curve which take into account class imbalance.

### Model comparison and hyperparameters

We performed a model comparison using five-fold cross validation to select the best architecture and related parameters for each level 1 category (“Disposition,” “Role,” “Process,” and “Physiological effect”). The model’s general structure can be seen in Fig. 3a. All the models were trained using the same hyperparameters that are listed in Table 1.

**Table 1.** Hyperparameters.

Hyperparameters	Selected value
Number of convolutional layers	2
Node features	64
Attention heads	8
Hidden dimension	32
Number of epochs	20
Threshold	0.5
Learning rate	0.005
Weight decay	0.001

### Attention nodes

The GAT architecture offers a class called “Explainer” which allowed us to easily interpret some of the results of our study. The GAT architecture we used consists of eight heads which are composed of attention nodes. An attention node has the advantage, as compared to a regular node, of giving a different importance (or weight) to each of its neighbors. These different weights are useful for classification and pattern detection. Our model uses the max attention explainer algorithm, which selects the biggest weight across the different heads for a given node or bond. Min-max scaling was used across each metabolites’ weights to distinguish more distinct patterns.

### Ablation study

We conducted an ablation study on the graph architectures to investigate the importance of coordinates and ChemBERTa embeddings in the model’s classification.

For the 2D coordinates, we omitted the input matrix of size N×2 (Fig. 3a) reducing the size of the node features from N×(M+2) to N×M. These models were trained in the exact same manner as models including 2D coordinates.

To assess the importance of the ChemBERTa embeddings, we omitted them reducing the input size of the fully connected layers (Fig. 3a). These models were trained in the exact same manner as models including 2D coordinates.

## Results

### Model comparison

**Figure 4.**
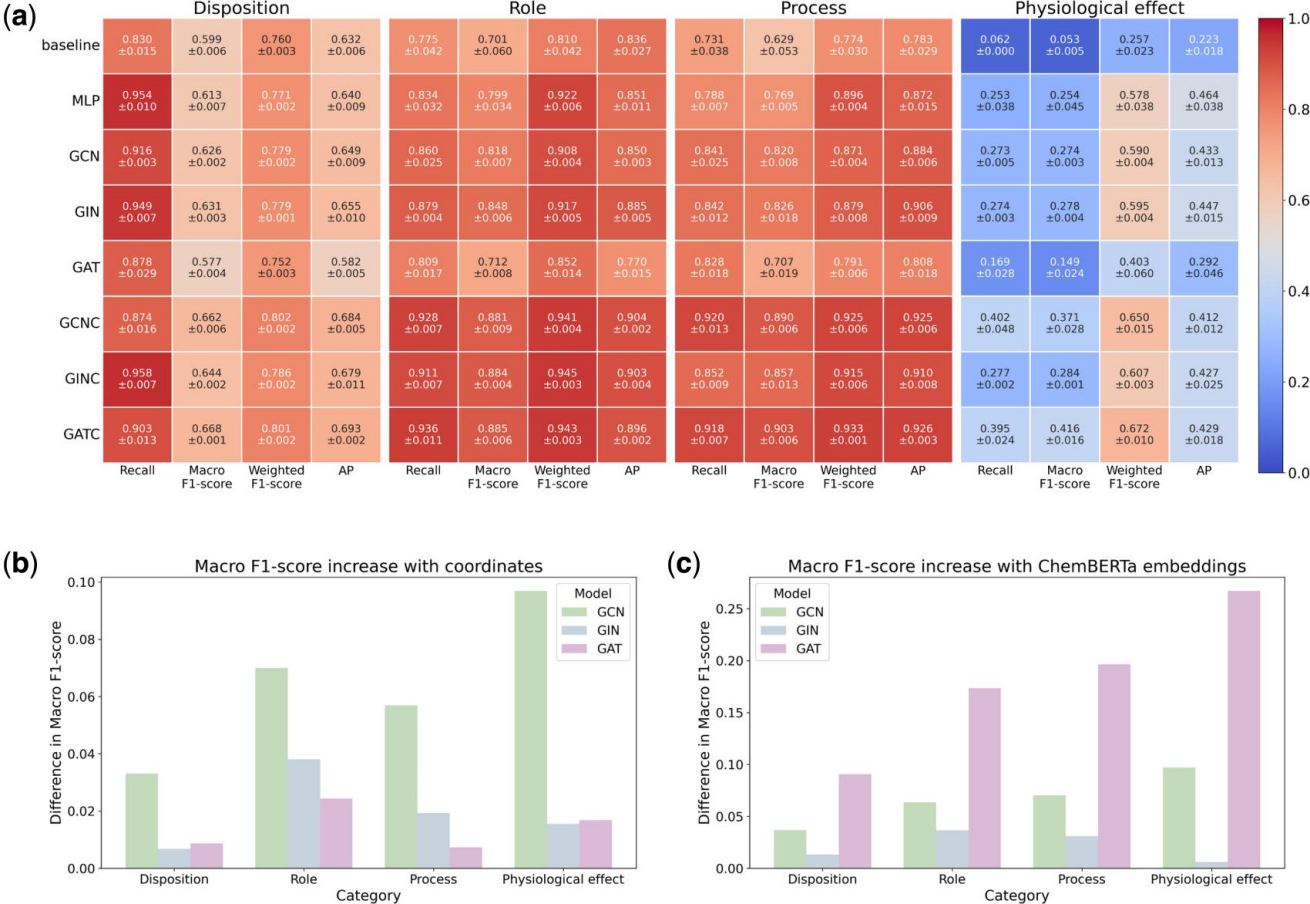
Visual representation of the results. (a) Each block corresponds to one category (from left to right: “Disposition,” “Role,” “Process,” and “Physiological effect”). Each column corresponds to a metric (from left to right in each block: recall, macro F1-score, weighted F1-score, and Average-Precision (also known as area under the precision-recall curve). Each row corresponds to a different model. The last three rows (denoted as GCNC, GINC, and GATC) respectively correspond to the GCN, GIN, and GAT architectures including ChemBERTa embeddings. Ablation study: (b) difference between models with or without coordinates. (c) difference between models with or without ChemBERTa embeddings.

Results of the comparison between the graph models across four categories can be seen in Fig. 4a. Overall, all models demonstrated strong performance across most categories, with the exception of the “Physiological effect” category, where performance was lower with a maximum macro F1-score of 0.416.

The majority of models outperform the baseline and the GAT architecture with ChemBERTa embeddings (GATC) is able to increase the macro F1-score by 0.363 over the baseline performance for the category “Physiological effect” (Fig. S3).

Graph models without ChemBERTa embeddings (rows labeled as “GCN,” “GIN,” “GAT” in Fig. 4a) perform similarly to the MLP model using only ChemBERTa embeddings (row labeled as “MLP” in Fig. 4a). The integration of graph models with ChemBERTa embeddings leads to improved performance.

GATC achieves the best macro F1-score for all categories, reaching 0.903 for the category “Process.” This consistently higher performance makes this model the best out of all eight we compared.

### Ablation study

To assess the contribution of different input features, we conducted an ablation study. In this section, we present the results based on the macro F1-score, while the results for other evaluation metrics are available in the supplementary material (Fig. S5).

We evaluated the importance of using 2D coordinates for the performance of the model (Fig. 4b). GCNs seem to benefit the most from the addition of coordinates with an average increase of 0.06 and a maximum increase of 0.09 for the category “Physiological effect.” A small increase is still observed for GINs (average increase of 0.02 in macro F1-score) and GATs (average increase of 0.02 in macro F1-score 0.01).

We also performed an ablation study for the ChemBERTa embeddings on the three graph models (Fig. 4c). Their addition improves results in all cases and seem to greatly help in the classification for the category “Physiological effect.”

### Interpretability

The use of attention nodes highlighted which bonds had the greatest influence on the model’s classification outcomes.

**Figure 5.**
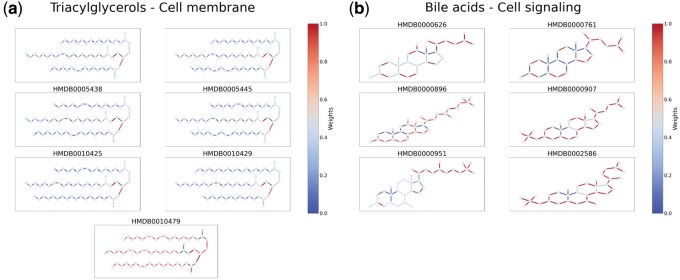
Interpretability analysis where bonds are colored based on the weight attributed by the model. Redder bonds are more important in the classification process. (a) Visual representation of triacylglycerol metabolites present in the test set for the classification of label “Cell membrane.” (b) Visual representation of bile acids present in the test set for the classification of label “Cell signaling.”

Our initial focus was on lipids, given their central role in cell membrane composition. Since lipids are key structural elements of membranes, the model is expected to recognize and represent this association in its predictions. We therefore trained a model following the same methodology as described earlier, specifically designed to classify whether a given metabolite is found in the cell membrane or not. This approach enabled us to develop a more specialized model and identify patterns in its learning process. In the test set for this model, 204 metabolites are labeled as lipids by the HMDB, and 151 of those are found in the cell membrane. The model correctly predicted cell membrane localization for 149 of these 151 metabolites, highlighting its accuracy in capturing this specific relationship. The model wrongly classified 16 metabolites (12 false positives and 2 false negatives) (Fig. S5). The use of attention nodes allowed us to assess bond-level importance in the classification process. Consistent patterns were observed among triacylglycerols, a lipid family predominantly found in cell membranes. Composed of three fatty acyl chains linked by a glycerol backbone (the lipid “head”), these molecules were primarily classified based on features near the head. As shown in Fig. 5a, attention weights (represented by bond shading) highlight a stronger focus on bonds near the head compared with those along the fatty acyl chains. However, in the case of the correctly classified metabolite HMDB0010479, this trend was less pronounced, with attention more evenly distributed.

A similar analysis of bile acids, in the context of predicting their association with cell signaling (Fig. 5b), showed that the model focused predominantly on bonds located at the periphery of the molecules, particularly around the side chains extending from the rigid steroid backbone. This attention pattern suggests that the classification relies on external structural features, which aligns with the biological role of bile acids in signaling pathways, where interactions typically occur at outward-facing regions involved in receptor binding.

## Discussion

Our study presents a novel way of combining different types of input and tackles multilabel classification which, to our knowledge, has not been previously performed for this dataset and task. We trained GNNs to predict multiple metabolite functions (location, role, process, and physiological effect) based on molecular structures.

We evaluated several commonly used graph-based architectures alongside two MLP models, including a baseline. The vast majority of graph-based models outperformed the baseline, supporting the effectiveness of incorporating molecular structure through graph representations and embeddings. Notably, the MLP model using only ChemBERTa embeddings achieved performance comparable with graph models that did not leverage these embeddings. The best results were obtained by combining graph-based architectures with ChemBERTa embeddings. An ablation study further underscored the significance of ChemBERTa embeddings in achieving high performance, and also revealed that including 2D molecular coordinates provides a modest performance gain. The small number of output nodes for “Process” and “Role” compared with the “Disposition” nodes could in part explain the higher performance on these categories in general, as multilabel prediction generally becomes more challenging as the number of labels increases. The model underperformed on the “Physiological Effect” task compared with the others, which could be because measurable physiological effects might be driven by differences in metabolite concentrations rather than just their presence and structure, on top of many additional possible confounders. Moreover, the small number of metabolites exhibiting “Physiological effect” nodes (Fig. S1) could also explain in part the poor performance.

No clear distinction in the HMDB is made between missing information and a given metabolite not having the function represented by a specific ontology term—there are no true negatives annotated in the dataset. This, combined with the general sparsity of annotations, contributes to the model having a large number of false-negative results.

In future work, we plan to tune the various hyperparameters of the architecture, such as the number of convolutional layers, to improve the performance of the model. An additional next possible step to improve the performance could be to use ChemBERTa token embeddings instead of averaging them as is currently done in our model. In this case, the model would use as input and as node feature for each atom and bond the exact atom or bond embedding given by ChemBERTa.

Kengkanna and Ohue (2024) introduced novel molecular graph representations by leveraging graph reduction techniques to enhance the capture of chemical substructures, including functional groups, chemical fragments, and pharmacophoric features. A promising avenue for future research lies in using these graph reduction methods to provide varying levels of detail about key characteristics relevant to compound property identification and interaction profiling, thereby potentially improving metabolite function prediction. Conformational flexibility in 3D space is one of the key characteristics that could be included in this way in future work.

The development of interpretability models in recent years has allowed for a better understanding of deep learning and for interpretation of various parameters. While we focused on *AttentionExplainer* (Fey and Lenssen 2019 ), many other models exist and could be leveraged to gain more insights into the classification process in future works.

Our model establishes the feasibility of broadly predicting functional ontology terms for metabolites based on their structural information. The availability of larger and more comprehensive datasets will be crucial for advancing the accuracy and applicability of machine learning techniques in this domain.

## Supplementary Material

vbaf174_Supplementary_Data

## Data Availability

All the code is available at https://github.com/TancrediCogne/MetaboliteGNN and the data needed to run the files can be downloaded directly on the HMDB website and on Zenodo.
