# Supplementary material for: Structure-based metabolite function prediction using graph neural networks
Source: Bioinform Adv. 2025 Jul 21;5(1):vbaf174. doi: 10.1093/bioadv/vbaf174 (PMC12343106; doi:10.1093/bioadv/vbaf174)
Supplement: vbaf174_Supplementary_Data [file vbaf174_supplementary_data.zip › Supplementary Material for Online Publication.pdf]

Supplementary Material

Structure-based metabolite function prediction using graph neural networks

Tancredi Cogne, Mariam Ait Oumelloul, Ali Saadat, Janna Hastings, Jacques Fellay

1 Data Parsing

To extract all the information about the metabolites, two files from the HMDB website were downloaded and used: `structures.sdf` and `hmdb_metabolites.xml`. The first one was used to extract the graph representation of each metabolite, i.e., the coordinates of each atom, the type of bonds, and atoms present in each bond. The second file was parsed to extract information such as SMILES, molecular weight, and ontology terms. The parsing of `hmdb_metabolites.xml` generated three files used in the project:

- `ontology_truth_table.csv`: each row is a binary vector signaling which ontology terms are present for a given metabolite
- `ontology_tree.csv`: the tree structure of the ontology with information such as the parent node and the node ID
- `metabolites_info.csv`: all information which is not related to ontology (taxonomy, molecular weight, SMILES, etc.)

These three files were used to process the data into the inputs and outputs described earlier.

2 Frequency of output labels

The barplots showing the number of 'Detected and quantified' metabolites exhibiting a chosen label can be seen in Fig. 4.

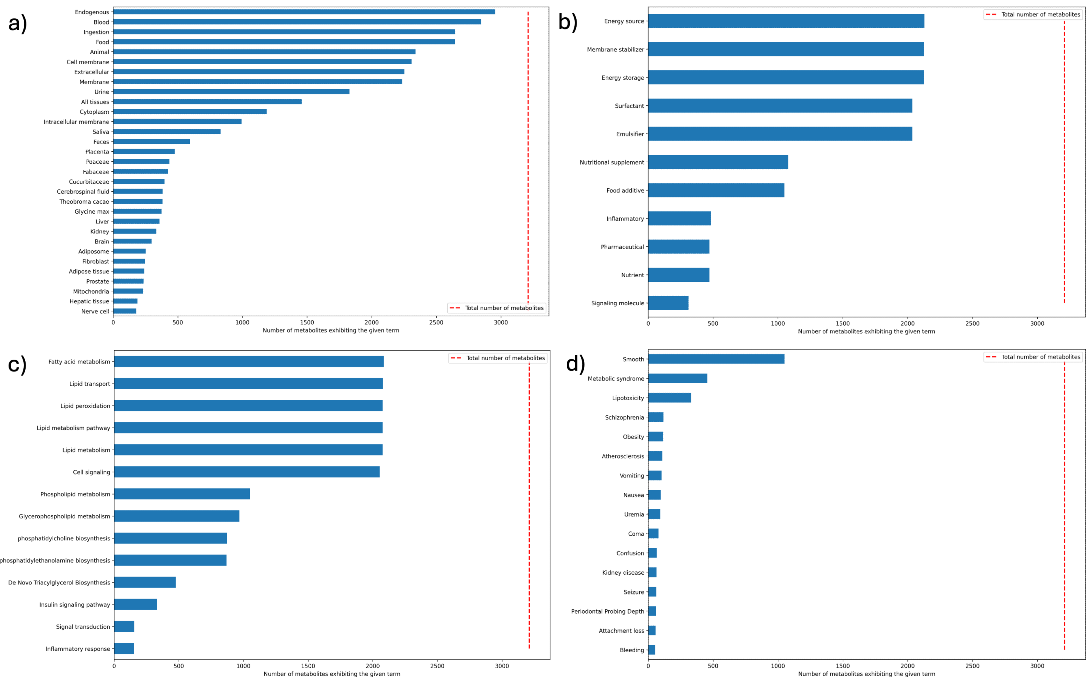

Figure 1: Number of metabolites exhibiting a chosen label. A red vertical doted line indicates the total number of metabolites (3,278). a) 31 labels of category 'Disposition' b) 11 labels of category 'Role' c) 14 labels of category 'Process' d) 16 labels of category 'Physiological effect'.

3 Architectures Mathematical Background

In this section, the mathematical basis of the three architectures used is described:

- Graph Convolutional Network (GCN):

$$H^{(l+1)} = \sigma\left(\tilde{D}^{-\frac{1}{2}}\tilde{A}\tilde{D}^{-\frac{1}{2}}H^{(l)}W^{(l)}\right)$$

  - $H^{(l)}$: Node feature matrix at layer l
  - $H^{(l+1)}$: Updated node feature matrix at layer $l+1$
  - $\tilde{A}$: Adjacency matrix A of the graph with added self-loops (identity matrix I_N)
  - $\tilde{D}$: Degree matrix of $\tilde{A}$
  - $\sigma(\cdot)$: Non-linear activation function such as ReLU or tanh
  - $W^{(l)}$: Weight matrix at layer l

- Graph Isomorphism Network (GIN):

$$H^{(l+1)} = \mathrm{MLP}^{(l)}\left(\left(1+\epsilon^{(l)}\right)H^{(l)} + \sum_{u\in\mathcal{N}(v)} H_u^{(l)}\right)$$

  - $H^{(l)}$: Node feature matrix at layer l
  - $H^{(l+1)}$: Updated node feature matrix at layer $l+1$
  - $\mathrm{MLP}^{(l)}$: Multi-layer perceptron applied at layer l
  - $\epsilon^{(l)}$: Learnable parameter (or constant)
  - $\mathcal{N}(v)$: Neighborhood of node v

- Graph Attention Network (GAT):

$$h_i^{(l+1)} = \sigma\left(\sum_{j\in\mathcal{N}(i)} \alpha_{ij}^{(l)} W^{(l)} h_j^{(l)}\right)$$

$$\alpha_{ij}^{(l)} = \frac{\exp\left(\mathrm{LeakyReLU}\left(a^T\left[W^{(l)}h_i^{(l)} \| W^{(l)}h_j^{(l)}\right]\right)\right)}{\sum_{k\in\mathcal{N}(i)}\exp\left(\mathrm{LeakyReLU}\left(a^T\left[W^{(l)}h_i^{(l)} \| W^{(l)}h_k^{(l)}\right]\right)\right)}$$

  - $h_i^{(l)}$: Feature vector of node i at layer l
  - $h_i^{(l+1)}$: Updated feature vector of node i at layer $l+1$
  - $\alpha_{ij}^{(l)}$: Attention coefficient between nodes i and j
  - $W^{(l)}$: Weight matrix at layer l
  - a: Learnable attention vector
  - $\|$: Concatenation operator
  - $\mathcal{N}(i)$: Neighborhood of node i
  - $\sigma(\cdot)$: Non-linear activation function such as ReLU or tanh

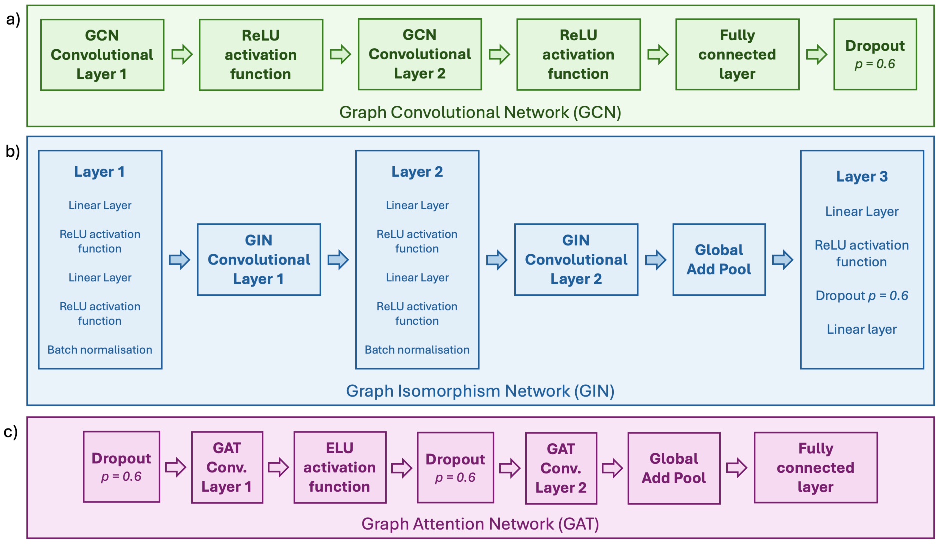

Figure 2: Summary of the tested graph architectures. a) Graph Convolutional Network (GCN) b) Graph Isomorphism Network (GIN) c) Graph Attention Network (GAT).

4 Difference in metrics compared to baseline

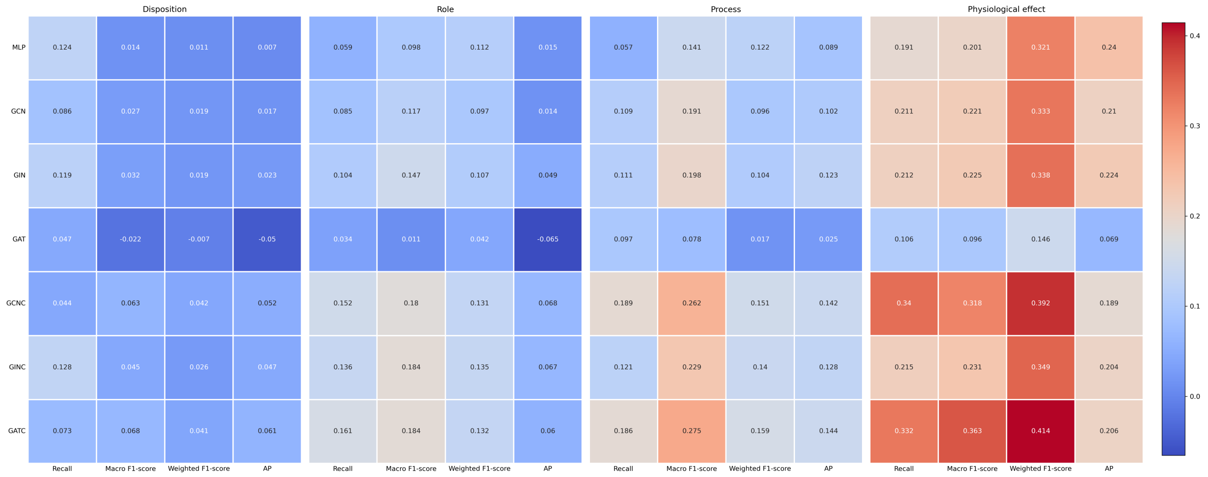

Figure 3: Visual representation of the difference between each model compared to baseline. Each block corresponds to one category (from left to right: 'Disposition', 'Role', 'Process', and 'Physiological effect'). Each column corresponds to a metric (from left to right in each block: recall, macro F1-score, weighted F1-score, and average-precision (also known as area under the precision-recall curve)). Each row corresponds to a different model. The last three row (denoted as GCNC, GINC, and GATC) respectively correspond to the GCN, GIN, and GAT architectures including ChemBERTa embeddings.

5 Ablation study

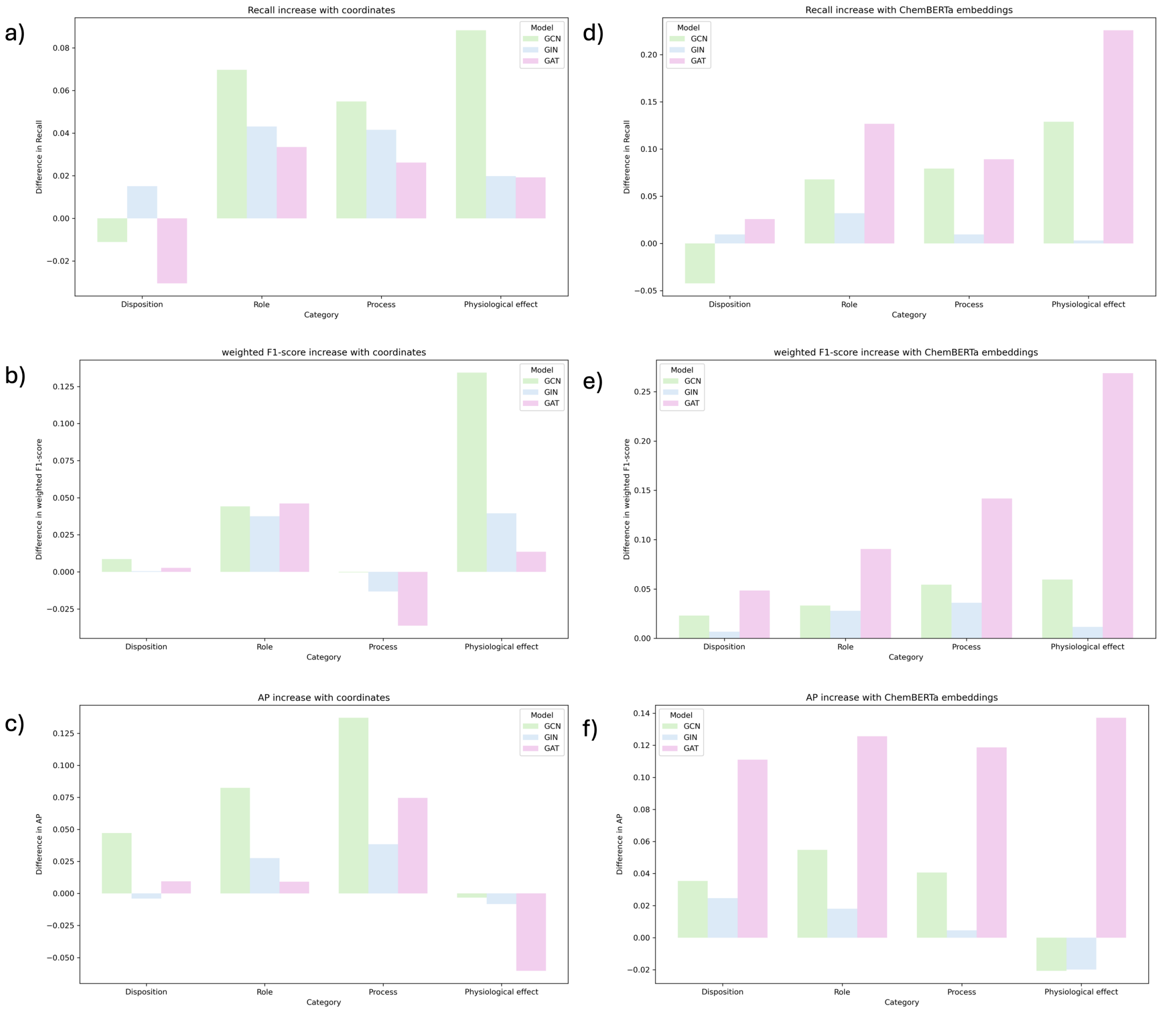

Figure 4: Visual representation of the increase (or decrease) in performance when using coordinates (subplots a), b), and c)) or ChemBERTa embeddings (sub-plots d), e), and f)).

6 Association between lipid metabolites and "Cell membrane" predictions.

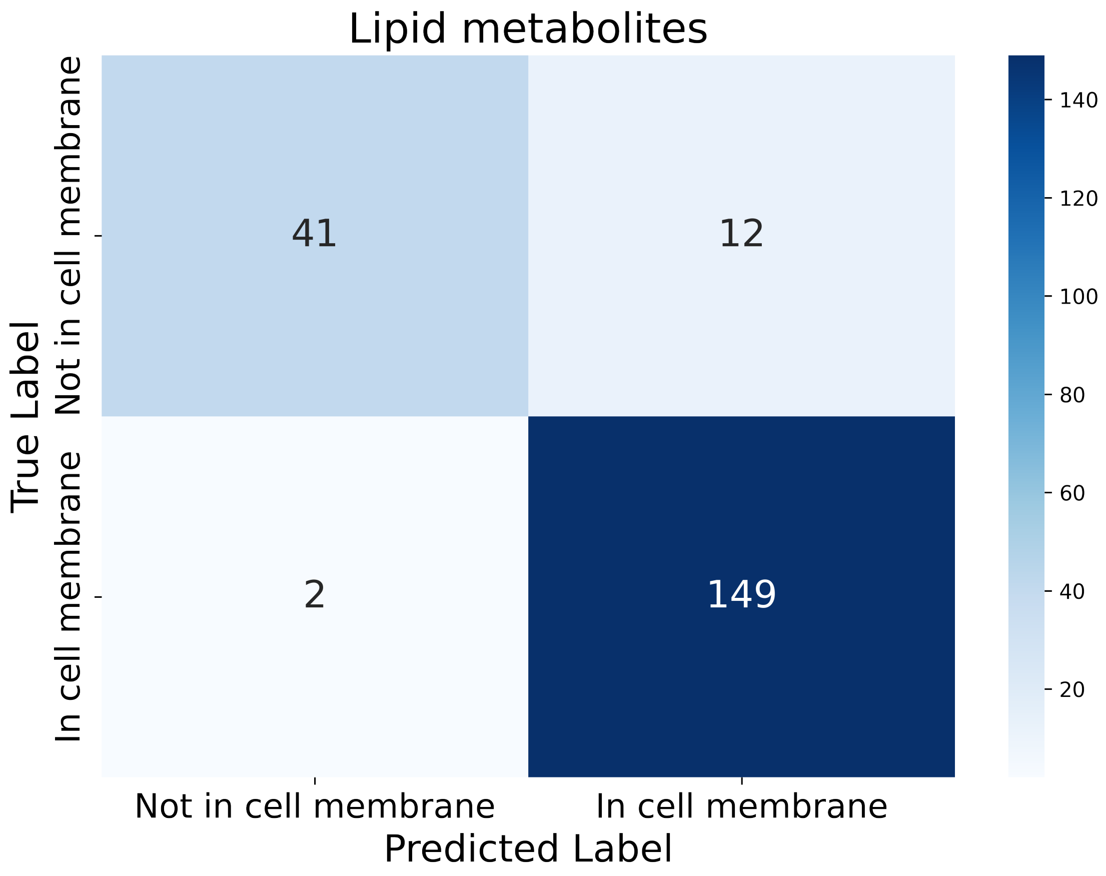

Figure 5: Heat map for the confusion matrix of "Cell membrane" predictions for lipid metabolites..
